# Parkinson’s disease clinical milestones and mortality

**DOI:** 10.1038/s41531-022-00320-z

**Published:** 2022-05-12

**Authors:** Maria Camila Gonzalez, Ingvild Dalen, Jodi Maple-Grødem, Ole-Bjørn Tysnes, Guido Alves

**Affiliations:** 1 Department of Quality and Health Technology, Faculty of Health Sciences, University of Stavanger, Stavanger, Norway; 2 The Norwegian Centre for Movement Disorders, Stavanger University Hospital, Stavanger, Norway; 3 Centre for Age-Related Medicine, Stavanger University Hospital, Stavanger, Norway; 4 Section of Biostatistics, Department of Research, Stavanger University Hospital, Stavanger, Norway; 5 Department of Chemistry, Bioscience and Environmental Engineering, University of Stavanger, Stavanger, Norway; 6 Department of Neurology, Haukeland University Hospital, Bergen, Norway; 7 Department of Neurology, Stavanger University Hospital, Stavanger, Norway

**Keywords:** Parkinson's disease, Risk factors

## Abstract

Identification of factors predicting and driving mortality in PD is important for patient information, disease management, and design of future clinical trials. This study included newly diagnosed PD patients and normal controls (NC) from a population-based study with repeated assessments over a 10-year period. We used the Kaplan–Meier method to estimate survival, Cox proportional hazards regression models to identify baseline risk factors of mortality, and Cox regression models with time-dependent covariates to evaluate the impact of four clinical milestones of advanced PD (visual hallucinations, recurrent falls, dementia, and nursing home placement) on mortality risk. During the 10-year study, 65 (34.2%) of 190 patients and 25 (12.3%) of 203 NC died, with an unadjusted hazard ratio (HR) of 2.85 (95% CI 1.80–4.52) and a HR of 2.48 (95% CI 1.55–3.95) when adjusted for confounders, including comorbidities. Higher age, more severe motor impairment, and postural instability-gait difficulty (PIGD) phenotype were independent baseline predictors of mortality. Each clinical milestone alone more than doubled the risk of death and had a cumulative effect on mortality, with a HR of 10.83 (95% CI 4.39–26.73) in those experiencing all four milestones. PD patients have an increased mortality risk that is disease-related and becomes evident early during the course of the disease. While motor severity and PIGD phenotype were early risk factors of mortality, clinical milestones signaled a substantially increased risk of death later during the disease course, highlighting their potential significance in clinical disease staging and prognosis.

## Introduction

Although most studies report increased mortality in Parkinson’s disease (PD), the reported estimates vary substantially, and some studies even found that survival is unaltered^1–4^. Similar variability is observed for various mortality risk factors, including demographics and motor and non-motor symptoms. At the same time, important clinical milestones (visual hallucinations, recurrent falls, dementia, and nursing home placement) remain understudied^5–9^.

Previous studies have estimated the degree of increased mortality risk in PD to range from 0.9 to 3.8^1,2^. The lack of accurate estimates can, in part, be explained by heterogeneity in study design. For example, the use of non-representative cohorts may yield higher mortality estimates than observed in the general PD population, as hospital-based studies are more likely to recruit advanced or atypical cases. Further, PD commonly occurs in the elderly population in which chronic comorbidities are common, and few studies have accounted for comorbidities, which may be an important source of confounding.

Accurate survival estimates and identification of early and late risk factors are valuable for health care planning and to recognise clinical features that might impact survival in clinical trials. However, given the limitations of previous studies, further studies are warranted to evaluate the true impact of PD on mortality. Against this background, we aimed to investigate survival in a group of incident PD patients followed for ten years from diagnosis compared to controls with comparable comorbidity status. Further, we sought to establish the contribution of key clinical milestones of advanced PD to excess mortality.

## Results

### Baseline characteristics

We included 393 subjects in this study; 190 patients with PD and 203 NC. Their demographics and clinical baseline characteristics are shown in Table 1.

**Table 1:** Baseline characteristics of Parkinson’s disease patients and normal controls.

	PD *n* = 190	NC *n* = 203	*p* value
Male, *n* (%)	115 (60.5)	106 (52.2)	0.09
Age in years	68.4 (9.3)	66.8 (9.6)	0.06
Smoking pack-years median (IQR)	0.00 (IQR 0–8.2)	2.00 (IQR 0–13.1)	0.05
CCI, median (IQR)	0.0 (IQR 0–1)	0.0 (IQR 0–1)	0.44
Time from diagnosis to baseline in months, median (IQR)	1.3 (IQR 0.6–1.9)	–	–
UPDRS motor score	23.6 (11.4)	–	–
Motor subtype		–	–
PIGD, *n* (%)	81 (41.2)	–	–
Tremor, *n* (%)	89 (46.3)	–	–
Indeterminate, *n* (%)	22 (12.5)	–	–
Hoehn & Yahr stage, median (IQR)	2.0 (IQR 1.5–2.5)	–	–
MADRS score, median (IQR)	3.0 (IQR 1.0–8.0)	0.0 (IQR 0.0–1.0)	<**0.001**
MMSE score, median (IQR)	28.0 (IQR 27.0–29.0)	29.0 (IQR 28.0–30.0)	<**0.001**

Values are means (SD) if not otherwise indicated. Bold values are statistically significant p < 0.05.

*PD* Parkinson’s disease, *NC* normal controls, *CCI* Charlson Comorbidity Index, *UPDRS* unified Parkinson’s disease rating scale, *MMSE* mini-mental state examination, *MADRS* Montgomery–Åsberg depression rating scale, *PIGD* postural instability and gait disorder.

### Mortality in PD and controls

**Fig. 1:**
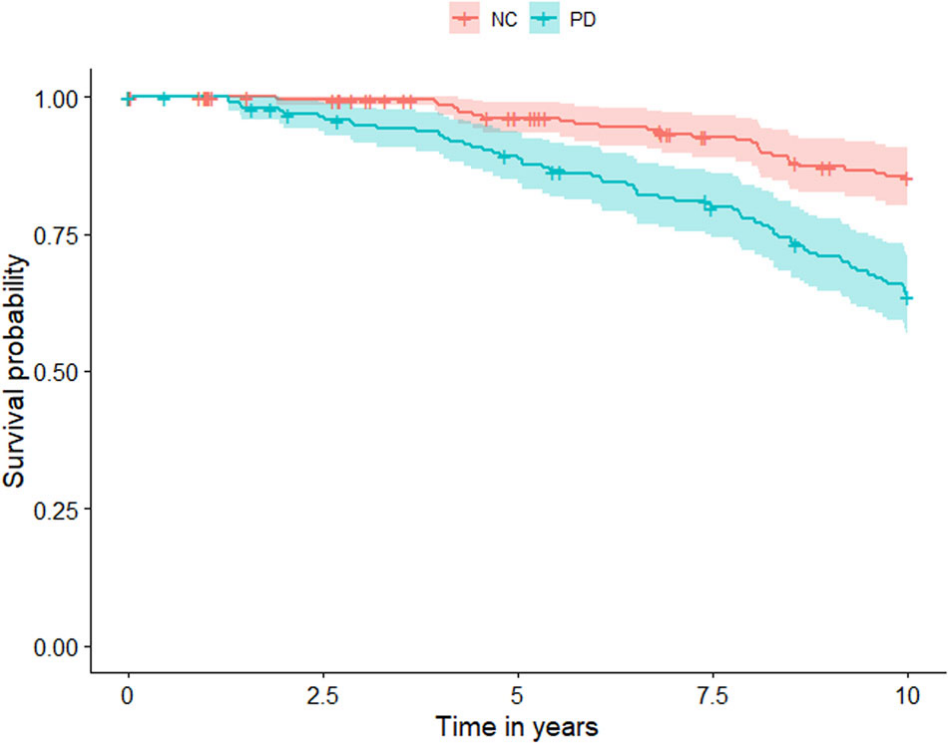
10-year survival in newly diagnosed Parkinson’s disease (PD) patients and normal controls (NC). Ten year survival curves of PD and NC are displayed here. The shaded area represents the confidence intervals.

During the study period, 65 (34.2%) of 190 patients and 25 (12.3%) of 203 NC died (Fig. 1). The risk of dying for PD patients was substantially increased during the first 10 years after diagnosis compared to controls, with hazard ratios (HR) of 2.85 (95% CI 1.80–4.52, *p* < 0.001) in the unadjusted model and 2.48 (95% CI 1.55–3.95, *p* < 0.001) after adjustment for potential confounders. The Cox regression analysis met the assumptions of proportional HRs (Supplementary Fig. 1).

### Baseline risk factors of mortality in PD

In addition to increased age (HR 2.20; 95% CI 1.52–3.17, *p* < 0.001), higher UPDRS motor scores (HR1.02; 95% CI 1.01–1.05, *p* = 0.02) and PIGD motor phenotype (HR 1.83; 95% CI 1.09–3.01, *p* = 0.02) were independent baseline predictors of mortality in PD. Neither sex, smoking, baseline HY stage, MMSE score, MADRS score, nor Charlson Comorbidity Index (CCI)^10^ predicted mortality among the PD patients (all *p* > 0.05, Supplementary Table 1).

### Clinical milestones and mortality in PD

A total of 89 PD patients experienced hallucinations within the 10-year follow-up, with a median time from baseline to this milestone of 4.9 years (IQR 2.2–7.0). Similarly, a total of 88 patients experienced recurrent falls and for these patients, the median time from baseline was 6.0 years (IQR 3.7–8.1). Among those who developed PDD, the median time from baseline to dementia diagnosis was 5.1 years (IQR 4.5–8.0). Lastly, for those admitted to a nurse-staffed long-term care facility (*n* = 43), the median time to admittance from baseline was 6.4 years (IQR 4.5–7.6).

**Fig. 2:**
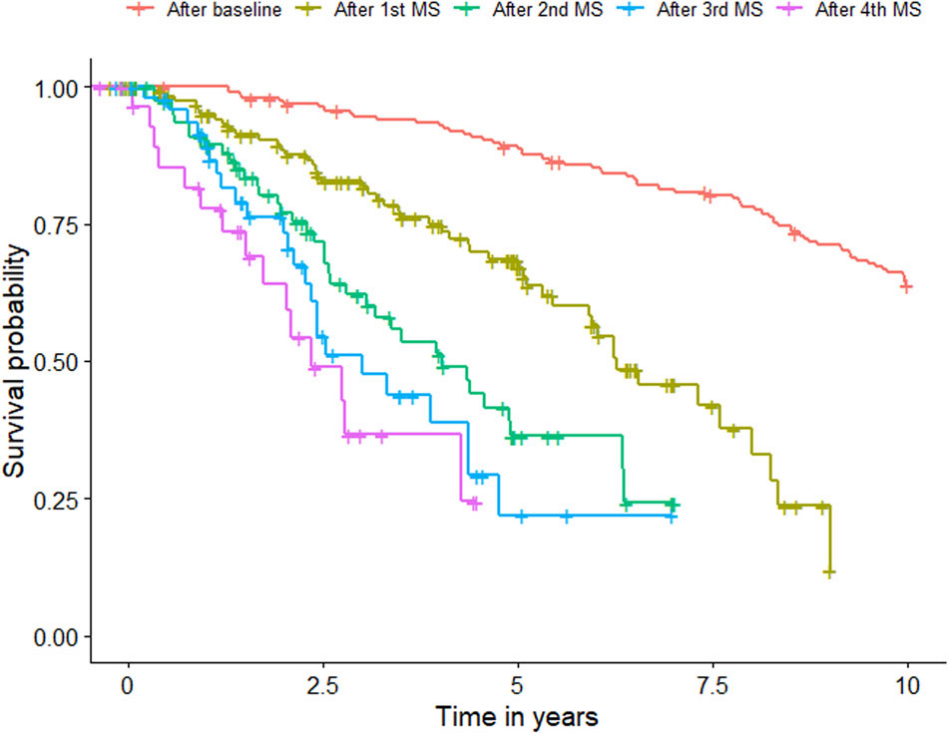
Kaplan–Meier curves for survival after cumulative clinical milestones occurrence. Here we display the survival curves for the presentation of clinical milestones (MS) cumulatively. The red curve represents the complete cohort’s survival and is included as a reference. The curve labels represent the cumulative presentation of the clinical milestones.

Falls and hallucinations were the clinical milestones with the longest time between occurrence and death, followed by the development of PDD, and admission to a nursing facility (Table 2). Cox regression models revealed that each of these milestones was associated with a substantially increased risk of dying when compared to those who did not experience the milestone (Table 2). Further analyses demonstrated that the risk of dying increased substantially with the cumulative number of milestones evolving over time (Table 2, Fig. 2). While the risk of dying during follow-up was more than doubled after the first event (HR 2.24; 95% CI 1.02–4.93, *p* = 0.043), it was about fourfold increased after the second and third events, and more than 10-fold increased for those developing all four event milestones (HR 10.83; 95% CI 4.39–26.73, p < 0.001).

**Table 2:** Clinical milestones as time-dependent covariate risk factors and time to death.

	Time to death, median (IQR)	Adjusted HR (95% CI)	*p* value
Falls	6.2 (3.6–8.9)	3.58 (2.02–6.33)	**<0.001**
Hallucinations	6.0 (4.3–7.7)	2.16 (1.26–3.71)	**0.004**
PDD	4.4 (3.6–5.1)	2.86 (1.59–5.13)	**0.004**
NHP	2.8 (2.3–3.2)	3.03 (1.65–5.52)	**<0.001**
*Cumulative clinical milestones*			
One clinical milestone	6.2 (5.9–8.2)	2.24 (1.02–4.93)	**0.043**
Two clinical milestones	4.0 (3.0–6.3)	4.04 (1.78–9.18)	**<0.001**
Three clinical milestones	2.9 (2.3–NA^a^)	4.53 (1.76–11.62)	**0.002**
Four clinical milestones	2.3 (1.7–NA^a^)	10.83 (4.39–26.73)	**<0.001**

Bold values are statistically significant. p < 0.05.

*PDD* Parkinson’s disease dementia, *NHP* nursing home placement, *IQR* interquartile range, *HR* hazard ratio, *CI* confidence interval.

^a^More than 25% of patients who had experienced this many milestones were still alive at end-of-study.

## Discussion

In this prospective population-based study of an incident PD cohort, we found excess mortality linked to PD that was evident early during the disease and more than doubled the risk of dying over the 10-year follow-up period independently of age, sex, smoking, and comorbidity status. While motor severity and phenotype were early disease-related risk factors of mortality in PD, the development of clinical milestones signaled a substantially increased risk of death later during the disease course. Thus, evaluating these clinical milestones may represent a meaningful way of assessing disease stage and prognosis in PD.

Other incident PD cohorts have reported a similar hazard rate for mortality in PD while adjusting for comorbidities, with slightly more than double the risk of death in patients with PD, but each of these cohorts included only one sex due to the original design of their studies^11,12^. Further, medical records–based studies have also reported similar risk while adjusting for the same confounders^13,14^ as well as captured how this risk gradually increases with advancing disease^4^. In contrast, the risk of death observed in our study is higher compared with the meta-analysis of other incident PD cohorts^1^. This could be related to the adjustment for comorbidities in our model, which could have uncovered a more substantial effect of PD on mortality than reported by prior studies. Additional factors contributing to the discrepancies in the reports of mortality risk attributed to PD in the literature are related to the different methods used to report risk^15,16^.

We showed that differences in mortality became evident early during the course of PD, which contradicts some studies of early PD suggesting that patients are not at increased risk of mortality until the later disease stages. Failure to detect the risk of mortality associated with PD in these studies might instead be explained by a study’s low statistical power or by recruiting an unbalanced control group in comorbidities, reducing the possibility of detecting the actual effect.

Previous studies have shown that life expectancy is lower in atypical parkinsonism than in PD patients^3^, however in our study the long follow-up period and rigorous diagnostic procedures make it unlikely that we have included atypical parkinsonism cases in our cohort; a claim that is further supported by our post-mortem findings in a substantial subset of patients. In our study, those with higher motor severity and PIGD phenotype are at the highest risk of early death and probably drive the early differences seen on a group level. Motor impairment and PIDG phenotype have similarly been associated with more deleterious forms of PD and more severe changes in both dopaminergic and non-dopaminergic brain structures^17^. Although the results of our study showed that motor symptoms predicted long-term survival at baseline, non-motor symptoms, assessed with the MMSE and MADS scales, and comorbidities were not associated with mortality. The latter finding indicates that the increased mortality risk in PD is disease-related and not linked to comorbidities at baseline.

Reaching PD clinical milestones has also been associated with more severe forms of PD using different clinical subtyping systems and developing dementia specifically with a higher risk of mortality^18–20^. In this study, the presence of any of these clinical milestones over time was associated with an at least 2-fold higher risk for mortality among PD patients.

Furthermore, we were able to observe a cumulative effect in the appearance of clinical milestones, with a HR of more than 10 in those experiencing all four milestones. In general, falls and hallucinations were the clinical milestones with the longest time between onset and death, followed by PDD development and admission to a nursing facility. This same sequential order of appearance supports the argument of a possible correlation between clinical features of the advanced disease state and the scores for cortical Lewy bodies^21^. Evaluating these clinical milestones may represent an alternative way of assessing advanced PD, since other traditionally used methods, such as the Hoehn and Yahr scale, are heavily based on motor disability, depreciating the multifaceted nature of the disease^22,23^.

Although this study is population-based and representative of the Norwegian population, not all of our results might be generalized to populations with different backgrounds, including demographics, comorbidities, and access to healthcare. However, this limitation is not unique to our study and does not affect the accuracy of the risks reported here. Furthermore, our study has several major strengths, including the population-based controlled design, frequent and standardized assessments from time of diagnosis, ten years of prospective follow-up, and the aforementioned post-mortem agreement of the diagnosis in a noteworthy subset of patients with PD.

This study provides essential data on mortality in PD and the close relationship between the clinical milestones and mortality risk, highlighting their potentially significant implications for clinical disease staging and prognosis.

## Methods

### Study design and population

The current investigation is part of the ongoing Norwegian ParkWest project, a prospective, population-based study of the incidence, neurobiology, and prognosis of PD^24^. The recruitment period was between 1 November 2004 and 31 August 2006. The recruitment methods and study design have been described before in detail^24^. In total, 212 incident PD cases and 205 controls assented to engage in the ParkWest study. Twenty-four participants were later excluded due to re-diagnosis (22 patients and 2 normal controls (NC)), leaving 393 eligible participants for this study. All patients fulfilled UK Brain Bank criteria^25^ of PD at their last interview, with a post-mortem agreement in a subgroup of patients who underwent autopsy (*n* = 42). Controls were recruited in the same geographical area from multiple sources, including friends and spouses of patients and members of public organizations for the elderly ensuring a similar socioeconomic background^26^.

### Standard protocol approvals, registrations, and patient consents

The study was approved by the Western Norway Regional Committee for Medical and Health Research Ethics (#2010/1700) and conducted according to the Declaration of Helsinki. All participants gave written informed consent.

### Examination program

We obtained demographics and medical history information with semi-structured interviews and performed comprehensive medical and neurological examinations at baseline. Standardized clinical follow-up was conducted every 6 months in patients, with extended visits in both patients and NC after 1-year and at 2-year intervals after that.

We rated disease stage at baseline and follow-up using the Hoehn and Yahr scale and motor severity using the unified PD rating scale (UPDRS) motor score (part III)^27,28^. Patients were assessed drug-naive (97.3%, *n* = 185) or in off-state at baseline, while in “on-state” during follow-up. The motor phenotype was classified as postural instability and gait disorder (PIGD) or tremor-dominant or intermediate following the classification algorithm described by Jankovic et al.^29^. We assessed global cognitive function using the mini-mental state examination (MMSE)^30^, neuropsychiatric symptoms using the 10-item neuropsychiatric inventory (NPI),^31^ and depressive symptoms using the Montgomery–Åsberg depression rating scale (MADRS)^32^.

For the present study, we evaluated clinical milestones as follows: visual hallucinations were deemed present when scoring ≥2 on UPDRS I item 2 (“benign” hallucinations with insight retained or worse) or ≥1 on NPI item 2 (presence of hallucinations). Recurrent falls were designated as obtaining a score of ≥2 on UPDRS II item 13 (occasional falls, less than once daily or worse) or ≥3 on UPDRS II item 14 (occasionally falls because of freezing or worse). Dementia associated with PD was defined as fulfilling Movement Disorders Society diagnostic criteria at level II^33,34^, and nursing home placement as first time admitted to a nurse staffed long-term care facility. Timing of milestones was set to the date of the first visit with criteria fulfilled for visual hallucinations, recurrent falls, and dementia, whereas for nursing home placement, the date of admittance was used.

We evaluated comorbidity burden with the CCI, a commonly accepted scale in epidemiologic research that employs a specified list of the most significant chronic diseases with regard to death. Mortality status was obtained by continual follow-up, including a review of clinical notes.

### Statistics

Baseline characteristics of the cohort are reported using counts and proportions for categorical variables and means and standard deviations for continuous variables. Comparison between groups was made with Student’s *t*-tests, Mann–Whitney *U* tests and Chi-square tests as appropriate. Each milestone’s time to occurrence for those who experienced the milestone within the 10 years follow-up was presented as medians and interquartile ranges (IQR).

Survival time following the inclusion date was analyzed using the Kaplan-Meier estimator (to allow for censoring) and summarized using the median survival time (either IQR or 95% confidence interval) and Kaplan–Meier plots. We compared the risk of death in PD and NC using Cox proportional hazards models. We adjusted models for sex, and baseline age and comorbidities using the CCI total score without considering age to avoid double adjustment and a clinical quantification of cigarette smoking using pack-year. We estimated hazard risk with their 95% confidence interval (CI) and tested with Wald tests. The proportional hazards assumption was tested using log minus log plots.

To explore early mortality risk factors in PD, we again used Cox models. The model included sex and baseline values for age, CCI total score (without considering age), UPDRS part III, motor phenotype classification, the Hoehn and Yahr scale, MMSE, smoking pack-year exposure, and MADRS.

Similarly, to examine late-stage PD mortality risk factors, median (IQR) survival time from the onset of each clinical milestone and from the onset of the kth milestone was estimated using the Kaplan–Meier estimator. Subsequently, the clinical milestones were entered individually into Cox models as time-dependent variables with values zero before the occurrence of milestones and values of one from the time of occurrence. For the cumulative count of milestones, the count was updated (to k) at the time of occurrence of the kth milestone and treated as a categorical variable. The HRs were adjusted with sex and baseline values for age, CCI total score (without considering age), UPDRS part III, motor phenotype classification, the Hoehn and Yahr scale, MMSE, smoking pack-year, and MADRS.

We considered *p* < 0.05 statistically significant. All statistical analyses were performed using SPSS 23.0 (IBM Corp., Armonk, NY) and R version 4.0.2. Software.

### Reporting summary

Further information on research design is available in the Nature Research Reporting Summary linked to this article.

## Supplementary information

Reporting Summary Checklist

Supplementary material

## Data Availability

Qualified external researchers can request access to anonymized patient-level data, respecting patient informed consent, from the corresponding author on reasonable request.
