# Supplementary material for: Parkinson’s disease clinical milestones and mortality
Source: NPJ Parkinsons Dis. 2022 May 12;8:58. doi: 10.1038/s41531-022-00320-z (PMC9098431; doi:10.1038/s41531-022-00320-z)
Supplement: Supplementary file 1 — Reporting Summary Checklist [file 41531_2022_320_MOESM1_ESM.pdf]

nature portfolio

Corresponding author(s): Manuscript Number: NPJPARKD-01096R

Last updated by author(s): Mar 10, 2022

Reporting Summary

Nature Portfolio wishes to improve the reproducibility of the work that we publish. This form provides structure for consistency and transparency in reporting. For further information on Nature Portfolio policies, see our Editorial Policies and the Editorial Policy Checklist.

Statistics

For all statistical analyses, confirm that the following items are present in the figure legend, table legend, main text, or Methods section.

n/a	Confirmed	
☐	☒	The exact sample size (*n*) for each experimental group/condition, given as a discrete number and unit of measurement
☒	☐	A statement on whether measurements were taken from distinct samples or whether the same sample was measured repeatedly
☐	☒	The statistical test(s) used AND whether they are one- or two-sided<br>*Only common tests should be described solely by name; describe more complex techniques in the Methods section.*
☐	☒	A description of all covariates tested
☐	☒	A description of any assumptions or corrections, such as tests of normality and adjustment for multiple comparisons
☐	☒	A full description of the statistical parameters including central tendency (e.g. means) or other basic estimates (e.g. regression coefficient) AND variation (e.g. standard deviation) or associated estimates of uncertainty (e.g. confidence intervals)
☐	☒	For null hypothesis testing, the test statistic (e.g. *F*, *t*, *r*) with confidence intervals, effect sizes, degrees of freedom and *P* value noted<br>*Give P values as exact values whenever suitable.*
☒	☐	For Bayesian analysis, information on the choice of priors and Markov chain Monte Carlo settings
☒	☐	For hierarchical and complex designs, identification of the appropriate level for tests and full reporting of outcomes
☒	☐	Estimates of effect sizes (e.g. Cohen's *d*, Pearson's *r*), indicating how they were calculated

Our web collection on statistics for biologists contains articles on many of the points above.

Software and code

Policy information about availability of computer code

Data collection	*Provide a description of all commercial, open source and custom code used to collect the data in this study, specifying the version used OR state that no software was used.*
Data analysis	*Provide a description of all commercial, open source and custom code used to analyse the data in this study, specifying the version used OR state that no software was used.*

For manuscripts utilizing custom algorithms or software that are central to the research but not yet described in published literature, software must be made available to editors and reviewers. We strongly encourage code deposition in a community repository (e.g. GitHub). See the Nature Portfolio guidelines for submitting code & software for further information.

Data

Policy information about availability of data

All manuscripts must include a data availability statement. This statement should provide the following information, where applicable:

- Accession codes, unique identifiers, or web links for publicly available datasets
- A description of any restrictions on data availability
- For clinical datasets or third party data, please ensure that the statement adheres to our policy

Qualified external researchers can request access to anonymized patient-level data, respecting patient informed consent, from the corresponding author on reasonable request.

Field-specific reporting

Please select the one below that is the best fit for your research. If you are not sure, read the appropriate sections before making your selection.

☐ Life sciences ☒ Behavioural & social sciences ☐ Ecological, evolutionary & environmental sciences

For a reference copy of the document with all sections, see nature.com/documents/nr-reporting-summary-flat.pdf

Behavioural & social sciences study design

All studies must disclose on these points even when the disclosure is negative.

Study description	We have conducted a community based study to estimate the incidence of PD in four counties of Norway using careful diagnostic procedures and standardised assessments as part of a prospective long term study of the incidence, neurobiology and prognosis of PD.
Research sample	The Norwegian ParkWest study is a prospective longitudinal cohort study of patients with incident Parkinson's Disease from Western and Southern Norway. The study area comprises the four counties of Sogn and Fjordane, Hordaland, Rogaland and Aust-Agder, with a total population of more than 1 million inhabitants.
Sampling strategy	We sought to recruit all residents in the study area with incident PD identified during a 22 month period between 1 November 2004 and 31 August 2006. In an attempt to achieve total ascertainment of patients with incident PD from Western and Southern Norway, the following search strategies were applied: (i) hand searching of all referral letters to the participating study centres for symptoms possibly representing incident parkinsonism; (ii) notification of all other hospital departments and all general practitioners, including consulting physicians of nursing homes, geriatric care centres and other institutions for persons of older age, by regular mail before the start of the study and by email reminders twice during the screening period; (iii) cooperation with the only neurologist exclusively working outside the five hospitals within the study area; (iv) electronic screening of hospital databases for patients being diagnosed with PD for the first time during the screening period and for 3 months after to capture delays in coding; and (v) an electronic population screening of 43 716 individuals of all ages. In addition, general practitioner files and drug prescriptions were searched electronically for antiparkinsonian drugs, including trade names of levodopa, various dopamine agonists, COMT inhibitors and selegiline. Single and combined search terms and diagnostic codes were used. Records dating back to 1 year before the start of the study were considered for screening.
Data collection	A multiple step diagnostic procedure was applied to identify patients with suspected incident PD. Visit comprised assessment of the patient's disease history, significant comorbidity, drug history, current medication and a general neurological examination by a study neurologist in all subjects, and the Mini-Mental State Examination when cognitive impairment was suspected. All subjects who consented to long term study participation and met the broad provisional criteria for incident PD, defined as the unequivocal presence of at least two of the four cardinal motor signs, typical disease history with evidence of progressive parkinsonism, no dementia at the onset of parkinsonism and no severe atypical signs (severe postural instability or frequent falls; prominent autonomic, pyramidal, cerebellar features or eye movement disorders), were directly forwarded to more comprehensive baseline assessments. In cases in which a diagnosis of PD was difficult to determine at the screening visit because of multiple potential underlying causes ("uncertain PD"), reassessments were conducted before study entry. In addition, [123I]FP-CIT dopamine transporter imaging was available to aid in the differential diagnosis.
Timing	22 month period between 1 November 2004 and 31 August 2006.
Data exclusions	Subjects who presented features definitely excluding PD, such as pre-existing dementia, or those who were free from parkinsonism. Twenty-four participants were later excluded due to re-diagnosis (22 patients and 2 normal controls (NC)), leaving 393 eligible participants for this study.
Non-participation	Please see Supplementary document as a table with this information has been provided.
Randomization	This study did not used randomization

Reporting for specific materials, systems and methods

We require information from authors about some types of materials, experimental systems and methods used in many studies. Here, indicate whether each material, system or method listed is relevant to your study. If you are not sure if a list item applies to your research, read the appropriate section before selecting a response.

Materials & experimental systems

n/a	Involved in the study
☒	☐ Antibodies
☒	☐ Eukaryotic cell lines
☒	☐ Palaeontology and archaeology
☒	☐ Animals and other organisms
☐	☒ Human research participants
☐	☒ Clinical data
☒	☐ Dual use research of concern

Methods

n/a	Involved in the study
☒	☐ ChIP-seq
☒	☐ Flow cytometry
☒	☐ MRI-based neuroimaging

Human research participants

Policy information about studies involving human research participants

Population characteristics: Drug naïve newly diagnosed PD patients and Controls were recruited in the same geographical area from multiple sources, including friends and spouses of patients and members of public organizations for the elderly ensuring a similar socioeconomic background.

Recruitment: This is part of a drug naïve, population based, incident cohort with PD study with multiple sources of identification to achieve total case ascertainment.

Ethics oversight: The study was approved by the Regional Committee for Medical and Health Research Ethics, Western-Norway, University of Bergen, Bergen, Norway.. All personnel who were part of the study group received comprehensive training in diagnostic and evaluation procedures before the start of the study and thereafter twice yearly.

Note that full information on the approval of the study protocol must also be provided in the manuscript.

Clinical data

Policy information about clinical studies

All manuscripts should comply with the ICMJE guidelines for publication of clinical research and a completed CONSORT checklist must be included with all submissions.

Clinical trial registration: *Provide the trial registration number from ClinicalTrials.gov or an equivalent agency.*

Study protocol: *Note where the full trial protocol can be accessed OR if not available, explain why.*

Data collection: *Describe the settings and locales of data collection, noting the time periods of recruitment and data collection.*

Outcomes: *Describe how you pre-defined primary and secondary outcome measures and how you assessed these measures.*
