# Supplementary material for: Parkinson’s disease clinical milestones and mortality
Source: NPJ Parkinsons Dis. 2022 May 12;8:58. doi: 10.1038/s41531-022-00320-z (PMC9098431; doi:10.1038/s41531-022-00320-z)
Supplement: Supplementary file 2 — Supplementary material [file 41531_2022_320_MOESM2_ESM.docx]

**The authors have provided this supplementary material to give readers additional information about their work.**

***Gonzalez MC, et al. Parkinson’s disease clinical milestones and mortality***

**Supplementary table 1.** **Association of baseline variables with mortality in Parkinson’s disease**

| **Baseline independent risk factors** | | | |
| --- | --- | --- | --- |
|  | Adjusted HR | (95% CI) | **p value** |
| Age | **2.20** | (1.52 – 3.17) | **<0.001** |
| Sex (Male) | 0.76 | (0.44 – 1.32) | 0.36 |
| Smoking Pack-Years | 1.00 | (0.98 – 1.02) | 0.67 |
| Charlson comorbidity index | 1.05 | (0.82– 1.34) | 0.82 |
| UPDRS motor score | **1.02** | (1.01 – 1.05) | **0.02** |
| PIGD motor phenotype | **1.83** | (1.09 – 3.01) | **0.02** |
| Hoehn and Yahr scale | 0.84 | (0.44 – 1.62) | 0.61 |
| MMSE score | 0.95 | (0.86 – 1.04) | 0.29 |
| MADRS score | 0.98 | (0.93 – 1.04) | 0.56 |

Hazard ratios (HRs) with 95% confidence intervals (CIs) for all continuous variables were calculated per unit except for age (HR per 10 years).

Abbreviations: (UPDRS) Unified Parkinson's disease Rating Scale, **(PIGD) Postural Instability - Gait Difficulty**, (MMSE) Mini-Mental State Examination, (MADRS) **Montgomery–Åsberg Depression Rating Scale,** (HR)

**Supplementary figure 1. Log minus log survival plot to asses the proportional hazard assumption in Parkinson’s Disease (PD) and Normal control’s (NC) mortality.**


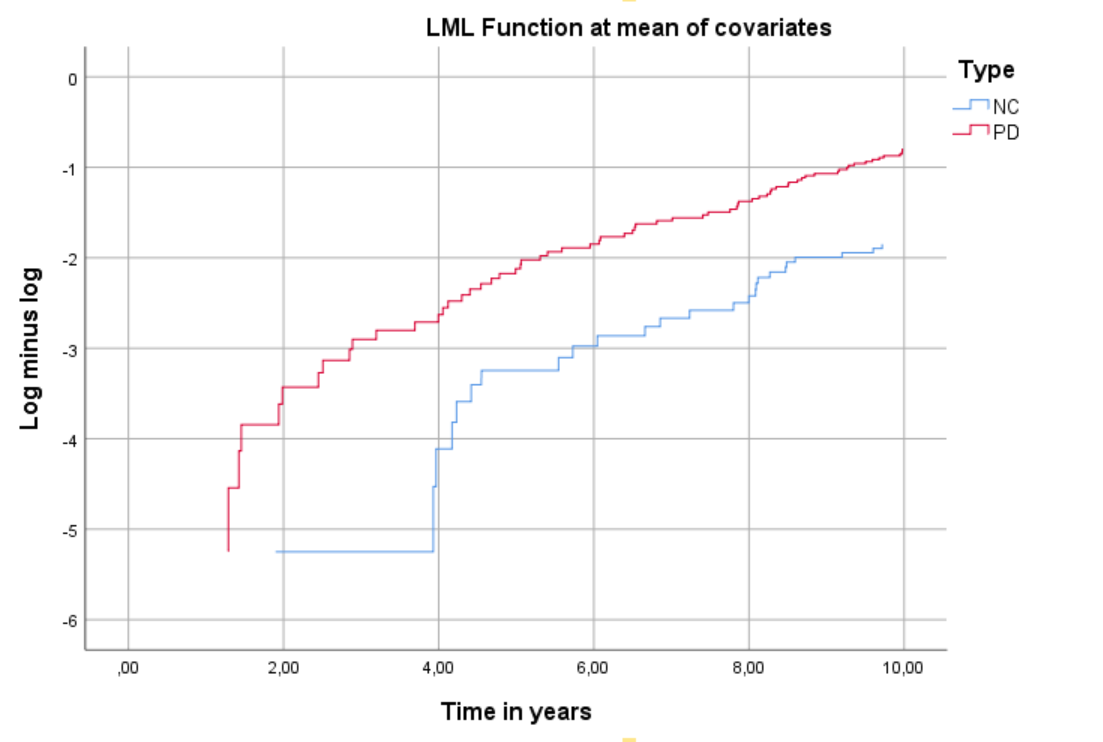


**Supplementary figure 2.** Flow chart of PD and NC participant dropouts.

**5-years visit**

159 patients

**3-years visit**

174 patients

**1-year visit**

186 patients

**Baseline**

190 patients

**4 drop-outs:**

1 dead

**12 drop-outs:**

9 dead

**15 drop-outs:**

13 dead

**15 drop-outs:**

12 dead

**7-years visit**

144 patients

**20 drop-outs:**

18 dead

**9-years visit**

124 patients

**25 drop-outs:**

23 dead

**10-years visit**

99 patients

**5-years visit**

169 NC

**3-years visit**

182 NC

**1-year visit**

192 NC

**Baseline**

203 NC

**11 dropouts:**

No deaths

**10 dropouts:**

1 dead

**13 dropouts:**

6 dead

**14 dropouts:**

5 dead

**7-years visit**

155 NC

**14 dropouts:**

11 dead

**9-years visit**

141 NC

**9 dropouts:**

5 dead

**10-years visit**

132 NC

**12 dropouts:**

9 dead

**13-years visit**

120 NC

**4 dropouts:**

4 dead
